# Attitude Towards Vaccination Among University Students at a Spanish University: Relationships with Sociodemographic and Academic Variables

**DOI:** 10.3390/vaccines12121301

**Published:** 2024-11-21

**Authors:** Francisco Javier Pérez-Rivas, Laura Esteban-Gonzalo, David García-García

**Affiliations:** 1 Grupo de Investigación UCM “Salud Pública-Estilos de Vida, Metodología Enfermera y Cuidados en el Entorno Comunitario”, Departamento de Enfermería, Facultad de Enfermería, Fisioterapia y Podología, Universidad Complutense de Madrid, 28040 Madrid, Spain; lesteb05@ucm.es (L.E.-G.); daviga27@ucm.es (D.G.-G.); 2 Red de Investigación en Cronicidad, Atención Primaria y Promoción de la Salud—RICAPPS—(RICORS), Instituto de Salud Carlos III, 28029 Madrid, Spain; 3 Instituto de Investigación Sanitaria Hospital 12 de Octubre (Imas12), 28041 Madrid, Spain; 4 Grupo de Investigación Cuidados Avanzados de Enfermería, Department of Nursing and Nutrition, School of Biomedical and Health Sciences, Universidad Europea de Madrid, 28670 Madrid, Spain

**Keywords:** attitudes towards vaccination, vaccine hesitancy, university students, sociodemographic factors, vaccination, anti-vaccination movement

## Abstract

Objectives: This descriptive, cross-sectional study examines the attitude towards vaccination of students at the Complutense University of Madrid (Spain) and explores its relationship with sociodemographic and academic variables using a bivariate analysis and linear and logistic regression. Methods: The attitude towards vaccination of 3577 students of different disciplines was assessed using an online version of the Questionnaire on Attitudes and Behaviours towards Vaccination. In addition, all students were asked if they sought information produced by anti-vaccination groups and whether they identified as “anti-vaccine”. Results: In general, the students showed a favourable attitude towards vaccination. Older students, those in paid employment, and those undertaking non-health-related studies had less favourable attitudes. Spanish-born and female students showed more positive attitudes than foreign-born and non-binary/male students, respectively. Only a small proportion of students identified as anti-vaccine. Conclusions: Despite these positive results, the need for interventions targeting specific groups with less favourable attitudes, such as older students, employed students, and those in non-health-related fields, is clear.

## 1. Introduction

Vaccination prepares the immune system for challenges by infectious diseases and has long had a crucial impact on public health. It is one of the most cost-effective and long-lasting of all health interventions; it reduces morbidity, mortality, and health inequalities worldwide and is key in protecting public health [1,2,3].

Despite their proven efficacy and overall safety, vaccines can cause mild side effects and, in rare cases, more serious adverse reactions, raising concern among certain sectors of the population [4]. This concern has been exacerbated by the spread of misinformation through digital channels, including the internet and instant messaging services, such as WhatsApp, Telegram, Instagram, or others, which has allowed anti-vaccination movements to gain unprecedented influence. These have sewn mistrust in, and indeed favoured the rejection of, vaccines among some members of the public. Effective responses need to be developed, rendering it critical to first explore the causes and contexts that fuel this mistrust and rejection [5,6].

Young people, particularly university students, consume large amounts of digital content every day, making them more susceptible to misinformation attacks, including on health-related topics [7]. The university years, which coincide with late adolescence and early adulthood, are a key period in the development of healthy behaviours and attitudes [8]. They are also a time when young people participate frequently in social activities and group events, share living spaces, and travel or work in direct contact with other people, increasing their potential exposure to infectious diseases [9]. While this population undoubtedly plays an important role in disease transmission, its number also includes future professionals who can directly influence health promotion and the implementation of health policies [10]. Their attitudes towards vaccination, therefore, could have a significant impact on public health.

Beliefs about and attitudes towards vaccination, which can be influenced by friends and family members, are key in vaccination acceptance and coverage rates. Certainly, lower vaccination rates have been associated with credence in conspiracy theories [5,11]. Moreover, the relationship between a person’s knowledge and vaccination decisions is not always clear or causal [12]. This highlights the need for more studies on attitudes towards vaccination in general. So far, most work has focused on the perceptions of specific target groups with respect to specific vaccinations, e.g., parents with respect to paediatric vaccination [13,14,15], adolescents with respect to COVID-19 [16,17], and HPV vaccination [18], pregnant women with respect to COVID-19 [19] and influenza vaccination [20], and oncology patients with respect to COVID-19 vaccination [21]. There is rather less information on the perceptions of adults in general with respect to COVID-19 [22,23,24] and HPV [25] vaccination. Numerous studies have also examined perceptions among students of health science [26,27,28,29,30,31,32] and among working health professionals [26,33,34,35,36,37,38]. However, studies on non-healthcare students are less common and have focused mainly on HPV [39,40,41], influenza [42], and COVID-19 [27,43,44,45,46,47] vaccination. This study aims to address this gap by examining attitudes towards vaccination among students of science, health sciences, arts, humanities, social sciences, engineering, and architecture at the Complutense University of Madrid (Spain), considering their academic level (bachelor’s, master’s, and doctoral) and their sociodemographic characteristics.

## 2. Material and Methods

### 2.1. Design and Participants

A descriptive, cross-sectional study was conducted with the aim of assessing the influence of sociodemographic and academic factors on the attitude towards vaccination of students enrolled at the Complutense University of Madrid (Spain), a public institution with over 65,000 students, offering a wide range of academic disciplines, including humanities, sciences, social sciences, health sciences, and engineering.

### 2.2. Sample Size Calculation

The sample size was calculated using the formula for estimating means in finite populations. With a population of 65,587 students at the Complutense University of Madrid, a standard deviation of 0.43 (based on a previous study on attitudes towards vaccination [6]), a 95% confidence level (z = 1.96), and a margin of error of 3%, it was estimated that at least 1729 students would be needed.

### 2.3. Procedures

Participants were recruited through the university’s Student Observatory (a service that analyses the needs and realities of the student body). For inclusion, they had to be enrolled in one of the degree courses offered by the university and have a sufficiently good understanding of Spanish to complete the questionnaire. After being contacted via institutional email and informed about the study’s objectives, all students who agreed to participate provided their informed consent electronically through an online checkbox at the beginning of the questionnaire, confirming their voluntary participation. Data were collected via an online questionnaire available between February and July 2024. The questionnaire was accessible through a link sent to the institutional email of the students. It was administered through Google Forms, restricted to users with valid university credentials, ensuring that only students could participate. The questionnaire was limited to a single response per participant to avoid duplicates. In total, responses were obtained from 3577 students, exceeding the calculated sample size, which ensures greater precision and reliability in the results obtained.

### 2.4. Study Variables

Attitudes towards vaccination were assessed via three indicators (here understood as dependent variables): (1) responses to the Questionnaire on Attitudes and Behaviours towards Vaccination (CAV) questionnaire; (2) the seeking of information produced by anti-vaccination groups; and (3) identifying as “anti-vaccine”.

- Questionnaire on Attitudes and Behaviours towards Vaccination. Developed and validated by Fernández-Prada et al. (2016) for use with Spanish university students, the self-administered CAV questionnaire examines beliefs, behaviours, and attitudes towards vaccination. Fifteen items were examined on a 5-point Likert scale (0 = strongly disagree, 4 = strongly agree). The scores for items 1, 2, 7, 8, 11, and 15, which were written in a negative sense, were converted to positive to align with the other items, and the total score was divided by the total number of items, providing a final score between 0 and 4. Higher scores reflect more favourable attitudes towards vaccination. The reliability of the scale in this study was adequate (Cronbach’s alpha = 0.89).
- Searching for information produced by anti-vaccination groups. All participants were asked: “Do you seek information produced by anti-vaccination groups?” (yes/no response options).
- Identification as anti-vaccine. This was assessed via the question: “Do you identify as “anti-vaccine”?” (yes/no response options).

The sociodemographic variables recorded were age, gender (male, female, non-binary, not willing to answer), country of birth (Spain, other), type of cohabitation (with friends/other students, with family, with partner, alone), and employment status (employed, unemployed). Information was also collected on the faculty in which each student was enrolled (arts and humanities, sciences, health sciences, engineering and architecture, and law and social sciences). Most bachelor’s degree programs at the university have a typical duration of 4 years. However, some programs, such as Medicine, Pharmacy, Veterinary Medicine, and certain dual-degree programs, require between 5 and 6 years to complete. Students were also asked whether they were studying for a bachelor’s, master’s, or PhD degree. In the case of master’s and PhD students, no information was requested on the academic year, as these studies have no defined year structure.

### 2.5. Statistical Analysis

Descriptive analyses were performed to highlight the characteristics of the sample. Quantitative variables are presented as mean and standard deviation, while qualitative variables are expressed as absolute and relative frequencies (percentages). Normality was assessed using the Kolmogorov–Smirnov test. Bivariate analyses seeking relationships between the three indicators of attitude towards vaccination and the studied sociodemographic/academic variables were performed using the following: (1) Pearson’s Chi-squared test to examine associations with qualitative variables; (2) the Mann–Whitney U and Kruskal–Wallis tests to examine associations with quantitative variables showing a non-normal distribution; and (3) Pearson’s correlation to examine associations with quantitative variables showing a normal distribution (Table 1).

Mixed-effects multilevel linear/logistic regression models were also used to analyse associations between the three indicators of attitude towards vaccines and the studied sociodemographic and academic variables. For CAV-other variable relationships, unadjusted linear models were used (Table 2); unadjusted logistic models were used for the other two indicators (Table 3 and Table 4). Subsequently, multilevel mixed-effects models were used to assess the influence of the branch of knowledge pursued, controlling for associated sociodemographic variables (Table 5). In all models, a random intercept was included for the student’s faculty and year (1st–6th), assigning a value of 0 for master’s and doctoral students. Finally, odds ratios (ORs) or Beta risk values were calculated, together with their standard errors, confidence intervals, and statistical significance (set at *p* < 0.05). All analyses were performed using the Statistical Package for the Social Sciences v.25 (SPSS. Inc., Chicago, IL, USA) or STATA/SE v.14.1 software (Stata Corp LP, College Station, TX, USA).

### 2.6. Ethics

The protocol of the present study was approved by the Research Ethics Committee of the Complutense University of Madrid (protocol code CE_20230413-11_SAL, approved on 13 April 2023). All participants were informed in writing about the aims and procedures of the research, and informed consent was requested before completing the questionnaire. Data were collected anonymously, ensuring confidentiality and compliance with current regulations.

## 3. Results

### 3.1. Description of the Sample and Analysis of Attitude Towards Vaccination

Of the 3577 students who completed the CAV questionnaire, 62.2% were female; the respondent mean age was 25.8 years (SD: 10.9). Most of the participants (85.4%) were born in Spain, 66.7% lived with their family, and 61.7% were not working at the time of the survey. A total of 71.5% were studying at the undergraduate level, mainly in the first or second year (54.6%) (Table 1).

The mean CAV score obtained was 3.10 out of 4 (SD: 0.67). Some 92% of the students reported that they did *not* seek information produced by anti-vaccination groups, while 97.9% did not identify as anti-vaccine (Table 1).

**Table 1:** Characteristics of University Students Participating in the Study on Vaccination Attitudes at the Complutense University of Madrid, Spain (2024).

*n*	3577	CAV Score [Mean, SD]	*P^a^*	Searches for Anti-Vaccine Group Info	*P^b^*	Identifies as ‘Anti-Vaccine’	*P^c^*
CAV score ^1^ (0–4) [mean, SD]	3.10 (0.67)						
Searches for information produced by anti-vaccination groups (%/*n*)			0.065 ^#^				**<0.001 ^z^**
Yes	8.0/286	2.8 (0.9)				47.3	
No	92.0/3291	3.1 (0.6)				52.7	
Identifies as ‘anti-vaccine’ (%/*n*)			**<0.001 ^#^**		**<0.001 ^z^**		
Yes	2.1/74	1.2 (0.5)		12.2			
No	97.9/3503	3.1 (0.6)		87.8			
Age [mean, SD]	25.8 (10.9)		**0.009 (−0.04) ^x^**	26.9 (11.4)	**0.005 ^#^**	27.7 (12.2)	0.076 ^#^
Gender (%)			0.002 *		**<0.001 ^z^**		**<0.001 ^z^**
Male	35.3	3.0 (0.7)		44.8		48.6	
Female	62.2	3.1 (0.6)		51.4		43.2	
Non-binary	1.6	3.1 (0.8)		3.5		8.1	
Does not wish to answer	0.8	2.9 (0.9)		0.3		0.0	
Country of origin (%)			**<0.001 ^#^**		0.751 ^z^		**0.018 ^z^**
Spain	85.4	3.1 (0.6)		86.0		75.7	
Other	14.6	2.9 (0.7)		14.0		24.3	
Cohabits with(%)			0.022 *		0.150 ^z^		**0.016 ^z^**
Friends/other students	14.9	3.1 (0.6)		13.6		12.2	
Family	66.7	3.1 (0.6)		62.9		59.5	
Partner	10.7	3.0 (0.7)		14.0		10.8	
Alone	7.7	2.9 (0.8)		9.4		17.6	
Employment status (%)			0.132 ^#^		0.185 ^z^		**<0.001 ^z^**
Currently employed	38.3	3.0 (0.7)		42.0		56.8	
Not currently employed	61.7	3.1 (0.6)		58.0		43.2	
Field of knowledge (%)			<0.001 *		0.469 ^z^		**<0.001 ^z^**
Arts and Humanities	19.3	3.0 (0.7)		23.1		27.0	
Sciences	20.7	3.2 (0.5)		19.9		9.5	
Health Sciences	29.2	3.2 (0.6)		29.0		16.2	
Engineering and Architecture	3.2	3.2 (0.6)		3.5		2.7	
Law and Social Sciences	27.6	2.9 (0.7)		24.5		44.6	
Type of studies (%)			0.011 *		0.546 ^z^		0.551 ^z^
Bachelor’s degree	71.5	3.1 (0.6)		69.9		77.0	
Master’s degree	17.5	3.0 (0.6)		17.1		14.9	
Doctoral studies (PhD)	11.0	3.1 (0.7)		12.9		8.1	
Year of study (bachelor’s studies only) (%)			0.100 *		**0.011 ^z^**		**0.003 ^z^**
1	31.7	3.1 (0.5)		22.8		18.3	
2	22.9	3.1 (0.6)		24.8		20.0	
3	17.9	3.0 (0.7)		19.4		25.0	
4	19.2	3.0 (0.7)		19.4		28.3	
5	6.4	3.2 (0.5)		9.7		1.7	
6	1.9	3.0 (0.8)		3.9		6.7	

^1^ CAV score= Questionnaire on Attitudes and Behaviours Towards Vaccination score. *P^a^* value relating CAV score and sociodemographic/academic variables. *P^b^* value relating searches for information produced by anti-vaccination groups and sociodemographic/academic variables. *P^c^* value relating identifying as anti-vaccine with sociodemographic/academic variables. ^x^ Spearman correlation test, *P* (correlation coefficient, r). # Mann–Whitney U-test. * Kruskal–Wallis test. ^z^ χ^2^ test. Bold values indicate statistically significant results (*p* < 0.05).

### 3.2. Bivariate Analysis: Attitude Towards Vaccination

Significant inverse associations were detected between the CAV score and identifying as anti-vaccine (*p* < 0.001) and age (*p* = 0.009, r = −0.04). Significant differences were also identified according to gender (*p* = 0.002), with women and non-binary people returning higher CAV scores, and according to country of birth (*p* < 0.001), with higher scores among students born in Spain. In addition, scores were higher among those living with friends/other students or family members (*p* = 0.022) and among science, health sciences, and engineering and architecture students (*p* < 0.001). Finally, undergraduate and PhD students showed more favourable attitudes towards vaccination than master’s degree students (*p* = 0.011).

### 3.3. Bivariate Analysis: Searching for Information Produced by Anti-Vaccination Groups

Seeking information produced by anti-vaccination groups was significantly associated with identifying as anti-vaccine (*p* < 0.001) and age (*p* = 0.005) (more common in older students). A higher proportion of males and non-binary individuals and a lower proportion of females sought anti-vaccine information (*p* < 0.001). A significant association was also found with academic year (*p* = 0.011), with information-seeking being more frequent among students in their final years of undergraduate studies, including the 5th and 6th years for those in longer programs such as Medicine, Veterinary Medicine, or Pharmacy).

### 3.4. Bivariate Analysis: Identifying as Anti-Vaccine

Students who identified as anti-vaccine showed more negative attitudes towards vaccination (*p* < 0.001). A higher proportion of males and non-binary individuals and a lower proportion of females identified as anti-vaccine (*p* < 0.001). Differences were also detected according to country of birth (*p* = 0.018), with a greater tendency among those born outside of Spain to identify as anti-vaccine and among those living alone (*p* = 0.016). Finally, employed people more often declared themselves anti-vaccine (*p* < 0.001), as did those studying social sciences, law, or arts and humanities (*p* < 0.001).

### 3.5. Multilevel Linear Regression: Attitude Towards Vaccination

Unadjusted multilevel linear regression models revealed that the most favourable attitudes towards vaccination (higher scores on the CAV questionnaire) were returned by female students (b = 0.08, *p* < 0.001), those born in Spain (b = 0.12, *p* < 0.001), and those studying science (b = 0.15, *p* = 0.036) or health science subjects (b = 0.17, *p* = 0.005). In contrast, less favourable attitudes were observed among older students (b = −0.002, *p* = 0.008), male students (b = −0.08, *p* < 0.001), students born outside Spain (b = −0.12, *p* < 0.001), students in paid employment (b = −0.04, *p* = 0.049), and among students of law and social sciences (b = −0.25, *p* < 0.001) (Table 2).

**Table 2:** Linear regression mixed models for Questionnaire on Attitudes and Behaviours towards Vaccination (CAV) score among University Students at the Complutense University of Madrid, Spain (2024).

		Model 1		
	*n*	β (SE)	95% CI	*P*
Age	3506	−0.002 (0.00)	−0.00–−0.00	**0.008**
Gender	3510			
Male	1235	−0.08 (0.02)	−0.13–−0.04	**<0.001**
Female	2187	0.08 (0.02)	0.03−0.13	**<0.001**
Non-binary	59	0.11 (0.08)	−0.05−0.28	0.175
Does not wish to answer	29	−0.16 (0.12)	−0.40−0.07	0.176
Country of origin	3510			
Spain	2999	0.12 (0.03)	0.05−0.18	**<0.001**
Other	511	−0.12 (0.03)	−0.18–−0.05	**<0.001**
Cohabits with	3510			
Friends/other students	528	0.02 (0.03)	−0.03−0.08	0.419
Family	2344	0.03 (0.02)	−0.00−0.08	0.108
Partner	376	−0.02 (0.03)	−0.09−0.04	0.544
Alone	262	−0.00 (0.02)	−0.05−0.04	0.814
Employment status	3510			
Currently employed	1338	−0.04 (0.02)	−0.09–−0.00	**0.049**
Not currently employed	2172	0.04 (0.02)	0.00−0.09	**0.049**
Field of knowledge	3510			
Arts and Humanities	669	−0.06 (0.08)	−0.23−0.10	0.046
Sciences	729	0.15 (0.07)	0.01−0.30	**0.036**
Health Sciences	1027	0.17 (0.06)	0.05−0.29	**0.005**
Engineering and Architecture	113	0.11 (0.16)	−0.19−0.43	0.462
Law and Social Sciences	972	−0.25 (0.04)	−0.34–−0.16	**<0.001**
Type of studies	3510			
Bachelor’s degree	2491	−0.01 (0.02)	−0.07−0.03	0.548
Master’s degree	626	−0.02 (0.03)	−0.08−0.03	0.442
Doctoral studies (PhD)	393	0.06 (0.03)	−0.01−0.13	0.095

Model 1: crude model. Bold values indicate statistically significant results (*p* < 0.05).

### 3.6. Multilevel Logistic Regression: Searching for Information Produced by Anti-Vaccination Groups

The use of unadjusted logistic mixed models showed that non-binary students were more likely to seek information produced by anti-vaccination groups (OR = 2.43, *p* = 0.013), followed by male students (OR = 1.58, *p* < 0.001). Females were less likely to seek such information (OR = 0.59, *p* = 0.008) (Table 3).

**Table 3:** Logistic regression mixed models for the condition of searching for information produced by anti-vaccine groups among University Students at the Complutense University of Madrid, Spain (2024).

		Model 1		
	*n*	OR	95% CI	*P*
Age	3506	1.00	0.99–1.01	0.110
Gender	3510			
Male	1235	1.58	1.23–2.04	**<0.001**
Female	2187	0.59	0.46–0.76	**<0.001**
Non-binary	59	2.43	1.20–4.90	**0.013**
Does not wish to answer	29	0.42	0.05–3.14	0.401
Country of origin	3510			
Spain	2999	1.04	0.73–1.49	0.802
Other	511	0.95	0.67–1.36	0.802
Cohabits with	3510			
Friends/other students	528	0.88	0.62–1.26	0.519
Family	2344	0.83	0.64–1.07	0.163
Partner	376	1.37	0.95–1.98	0.082
Alone	262	0.90	0.68–1.19	0.482
Employment status	3510			
Currently employed	1338	1.17	0.91–1.51	0.210
Not currently employed	2172	0.85	0.66–1.09	0.210
Field of knowledge	3510			
Arts and Humanities	669	1.32	0.91–1.91	0.140
Sciences	729	0.97	0.66–1.42	0.904
Health Sciences	1027	0.99	0.70–1.39	0.966
Engineering and Architecture	113	1.14	0.52–2.50	0.739
Law and Social Sciences	972	0.80	0.57–1.13	0.211
Type of studies	3510			
Bachelor’s degree	2491	0.94	0.70–1.25	0.684
Master’s degree	626	0.94	0.67–1.32	0.753
Doctoral studies (PhD)	393	1.19	0.81–1.73	0.357

Model 1: crude model. Bold values indicate statistically significant results (*p* < 0.05).

### 3.7. Multilevel Logistic Regression: Identifying as Anti-Vaccine

Logistic mixed model analysis showed that male (OR = 1.74, *p* = 0.025) and non-binary (OR = 6.47, *p* < 0.001) students were more likely to consider themselves anti-vaccine and that females were less likely to do so (OR = 0.45, *p* < 0.001). In addition, students in paid employment (OR = 2.31, *p* = 0.001) and students of law and social sciences (OR = 2.40, *p* = 0.010) were more likely to identify as anti-vaccine, while science (OR = 0.30, *p* = 0.037) students were less likely to do so (Table 4).

**Table 4:** Logistic regression mixes models for identifying as anti-vaccine among University Students at the Complutense University of Madrid, Spain (2024).

		Model 1		
	*n*	OR	95% CI	*P*
Age	3506	1.01	0.99–1.03	0.140
Gender	3510			
Male	1235	1.74	1.07–2.85	**0.025**
Female	2187	0.45	0.27–0.73	**0.001**
Non-binary	59	6.47	2.51–16.69	**<0.001**
Does not wish to answer	29	1.00	-	-
Country of origin	3510			
Spain	2999	0.58	0.32–1.04	0.068
Other	511	1.71	0.96–3.05	0.068
Cohabits with	3510			
Friends/other students	528	0.75	0.36–1.54	0.441
Family	2344	0.76	0.46–1.25	0.282
Partner	376	1.02	0.47–2.22	0.945
Alone	262	1.18	0.67–2.09	0.552
Employment status	3510			
Currently employed	1338	2.31	1.39–3.83	**0.001**
Not currently employed	2172	0.43	0.26–0.71	**0.001**
Field of knowledge	3510			
Arts and Humanities	669	1.43	0.56–3.69	0.448
Sciences	729	0.30	0.10–0.93	**0.037**
Health Sciences	1027	0.53	0.22–1.25	0.153
Engineering and Architecture	113	0.88	0.11–6.53	0.901
Law and Social Sciences	972	2.40	1.23–4.68	**0.010**
Type of studies	3510			
Bachelor’s degree	2491	1.38	0.67–2.81	0.370
Master’s degree	626	0.87	0.41–1.82	0.713
Doctoral studies (PhD)	393	0.74	0.30–1.84	0.525

Model 1: crude model. Bold values indicate statistically significant results (*p* < 0.05).

### 3.8. Multilevel Linear/Logistic Regression Adjusted for the Three Indicators of Attitude Towards Vaccination

The adjusted models for the three indicators of attitude towards vaccination confirmed the previously described associations, with significant relationships particularly well maintained between branch of knowledge and the CAV questionnaire score and identifying as anti-vaccine (Table 5).

**Table 5:** Linear/logistic adjusted regression mixed models for exploring the associations between the three indicators of attitude towards vaccination and field of knowledge among University Students at the Complutense University of Madrid, Spain (2024).

CAV Score		Model 1		
	*n*	β (SE)	95% CI	*P*
Field of knowledge	3510			
Arts and Humanities	669	−0.05 (0.08)	−0.21–0.11	0.530
Sciences	729	0.15 (0.07)	0.01–0.29	**0.029**
Health Sciences	1027	0.16 (0.06)	0.04–0.28	**0.008**
Engineering and Architecture	113	0.13 (0.15)	−0.17–0.43	0.402
Law and Social Sciences	972	−0.25 (0.04)	−0.33–−0.16	**<0.001**
Searching for information produced by anti-vaccine groups		**Model 2**		
	***n***	**OR**	**95% CI**	***P***
Field of knowledge	3510			
Arts and Humanities	669	1.34	0.92–1.95	0.125
Sciences	729	0.90	0.62–1.32	0.621
Health Sciences	1027	1.05	0.74–1.48	0.766
Engineering and Architecture	113	1.01	0.45–2.23	0.979
Law and Social Sciences	972	0.80	0.57–1.14	0.234
Considering oneself to be anti-vaccine		**Model 3**		
	***n***	**OR**	**95% CI**	***P***
Field of knowledge	3510			
Arts and Humanities	669	1.49	0.59–3.74	0.394
Sciences	729	0.29	0.09–0.89	**0.031**
Health Sciences	1027	0.55	0.23–1.30	0.177
Engineering and Architecture	113	0.84	0.11–6.08	0.866
Law and Social Sciences	972	2.30	1.19–4.44	**0.013**

Model 1: adjusted for age, gender, country of origin and employment status. Model 2: adjusted for gender. Model 3: adjusted for gender and employment status. Bold values indicate statistically significant results (*p* < 0.05).

## 4. Discussion

The present students generally showed a positive attitude towards vaccination, with a mean CAV score of 3.10 out of 4 (SD: 0.67), reflecting strong acceptance of vaccines. Only 8% of the students claimed to have sought information produced by anti-vaccination groups, and only 2.1% identified as anti-vaccine. These results contrast with previous studies, both in university students [26,43] and in the general population [22], in which lower percentages of acceptability were observed. The difference may lie in the fact that many of these studies focused on COVID-19 vaccination during the pandemic, when uncertainty, rapid vaccine development, and media coverage may have negatively influenced people’s perceptions of vaccination [48]. Studies on the HPV vaccine have returned better acceptance results [41]. In addition, Spain ranks among the countries with the highest acceptance of vaccines [22], which may be reflected in the observed results.

Attitude towards vaccination and anti-vaccine information-seeking were significantly associated with identifying as anti-vaccine (*p* < 0.001); this is consistent with that reported in the literature [5,11]. The relationship between attitude, information sources, and individual perception is well documented: exposure to anti-vaccination information may reinforce negative attitudes towards vaccines, creating a cycle of mistrust.

An inverse relationship was seen between attitude towards vaccination (*p* = 0.009, r = −0.04) and age (b = −0.002, *p* = 0.008). This contrasts with the results of other studies, in which the older population tended to show greater trust in the health care system and have higher vaccination rates [24,49,50]. However, the present results may reflect the specific composition of the university student sample; older students, who are less common, might be less exposed to vaccination campaigns targeting the university population or have a more fixed position on vaccines based on previous experiences. No relationship was seen between age and seeking anti-vaccine information; it may be that current widespread access to information is narrowing the gap between generations in terms of exposure to information sources.

Gender was an important factor in attitude towards vaccination, seeking anti-vaccine information, and identifying as anti-vaccine. Females returned higher CAV scores, and being female was a positive predictor of having a good attitude (b = 0.08, *p* < 0.001). Males had a more negative attitude (b = −0.08, *p* < 0.001) and were more likely to seek anti-vaccine information (OR = 1.58, *p* < 0.001). These findings contradict those of previous studies suggesting that women tend to be more concerned about side effects and show greater reluctance towards vaccination [24,48,51,52]. However, most of the available studies for comparison focus on COVID-19 vaccination, and differences are likely to vary depending on the type of vaccine and the social and cultural context of the study. The non-binary students were most likely to seek information produced by anti-vaccination groups (OR = 2.43, *p* = 0.013) and to identify as anti-vaccine (OR = 6.47, *p* < 0.001). This may reflect a higher perceived vulnerability within this minority group, which faces disparities in the health system. Its members may, therefore, feel more distrustful of vaccination [53].

Spanish-born students showed a more favourable attitude towards vaccination (*p* < 0.001) than foreign-born students. The latter, most of whom were from countries with a lower income than Spain’s, were more likely to identify as anti-vaccine (*p* = 0.018), suggesting that prior health education, socioeconomic, and vulnerability factors may influence their attitudes towards vaccination.

Students living with friends or peers scored higher on the CAV (*p* = 0.022). In addition, those living alone were more likely to consider themselves anti-vaccine (*p* = 0.008). This might be explained by a greater sense of isolation and mistrust compared to those living with others, who might feel more responsible for and discuss the health of those in their immediate environment.

Being a student with a paid job had a negative impact on attitude towards vaccination, reflected by a lower CAV score (b = −0.04, *p* = 0.049). In addition, the members of this group were more likely to identify as anti-vaccine (OR = 2.31, *p* = 0.001) compared to students with no paid employment (OR = 0.43, *p* = 0.001). The literature contains no information on this characteristic in university students, but it may be that the combination of study and work is stressful for students, in some way increasing mistrust in the health care system.

Field of knowledge also had an effect on attitudes towards vaccination. Students of the sciences, the health sciences, engineering, and architecture returned higher CAV scores (*p* < 0.001). In contrast, students of law and social science had less favourable attitudes (b = −0.25, *p* < 0.001) and were more likely to identify as anti-vaccine (OR = 2.40, *p* = 0.010). These findings are consistent with reports in the literature suggesting that students in health-related fields have more positive attitudes towards vaccination [2,39,43].

The CAV scores returned by the undergraduate and doctoral students were a small—but, in bivariate analysis, significant (*p* = 0.011)—0.1 points higher than those of the master’s degree students. In multiple regression analysis, however, this significance was not maintained. The lack of difference could be due to the fact that all the participants were university educated and that a high level of education improves attitudes towards vaccines [25,52]. Certainly, higher educational attainment is associated with higher levels of health literacy, which positively influences risk perception, confidence in vaccines, and willingness to be vaccinated [54,55]. However, seeking information produced by anti-vaccination groups was more prevalent among the 5th and 6th year students (*p* = 0.011), a result that contrasts with the findings of Sakamoto et al. [44], who reported a more positive attitude towards vaccines with academic year. However, it may be that the present 5th and 6th year students, who were largely studying health care subjects—mostly Medicine—and who are close to entering the labour market, felt a greater need to be better informed about vaccination misinformation. Students in their 3rd and 4th year (taking all subjects as a whole) were most likely to identify as anti-vaccine (*p* = 0.003). It may be that by this time, this population subset has been exposed to more information sources, generating a certain vaccination scepticism amongst some of its members.

The study suffers from a number of limitations. For example, its cross-sectional design precludes the establishment of any causal relationships. In addition, the research was conducted at a single university in Madrid; the results might differ at other institutions. Nonetheless, the work included a comprehensive analysis encompassing gender, field of knowledge, and employment status, providing valuable information on the relationship between these factors and attitude towards vaccination, associations that have been little explored.

## 5. Conclusions

The study population showed an overall favourable attitude towards vaccination, with only 2,1% of students identifying as anti-vaccine. This reflects strong support for vaccines in this population and a positive perception of vaccination. The proportion of students seeking anti-vaccine information was also limited (8%), indicating that most students are not influenced by today’s negative narratives surrounding vaccination.

Poor attitude towards vaccination and seeking anti-vaccine information were significantly related to identifying as anti-vaccine. In particular, older students, those in paid employment, and those studying law and social sciences had a less favourable attitude towards vaccination. In contrast, female students, the Spanish-born population, those who lived with friends or other students or family members, and students of the sciences and health sciences showed more positive attitudes.

Non-binary students, students who lived alone, those born outside Spain, or who were in paid employment were the most likely to identify as anti-vaccine. Female gender was associated with the lowest likelihood of being anti-vaccine. Health science students, especially those in the final years of their studies, were the most likely to seek information produced by anti-vaccination groups, but perhaps only to be better able to counter it.

Finally, attitudes and behaviour towards vaccines were little influenced by the level of studies undertaken (bachelor’s, master’s, or doctoral degree). This suggests that, given their high level of education, all university students share a similar perception of vaccination.

Despite these very positive results, it remains critical that immunisation be promoted, following strategies that address the concerns and mistrust of those groups that show the most negative attitudes. Interventions could include educational campaigns that address misconceptions about vaccines, workshops promoting critical appraisal of information sources, and peer-led discussions in which students from health-related fields become vaccine advocates. Additionally, flexible formats, such as online sessions and short informational videos, could be highly effective, especially for students with limited time availability. Another valuable initiative could be the creation of dedicated vaccination sections on university websites. These spaces could include educational materials debunking vaccine myths and evidence-based content to support informed decision-making among students.

## Data Availability

The data presented in this study are available upon request from the corresponding author.
